# One Approach Anterior Decompression and Fixation with Posterior Unilateral Pedicle Screw Fixation for Thoracolumbar Osteoporotic Vertebral Compression Fractures

**DOI:** 10.1111/os.12947

**Published:** 2021-03-30

**Authors:** Hui‐wang Wang, Yong‐cheng Hu, Zhan‐yong Wu, Hua‐rong Wu, Jian‐qing Ma, Hui‐qiu Jian, Sheng‐hua Ning, Wen‐Kun Xu

**Affiliations:** ^1^ Department of Orthopaedics Orthopaedic Hospital Xingtai China; ^2^ Department of Spinal Surgery Tianjin Hospital Tianjin China; ^3^ Department of Orthopaedic Laboratory Xingtai Institute of Orthopaedics Xingtai China

**Keywords:** Compression, Fracture fixation, Osteoporotic, Retrospective studies, Spinal fractures

## Abstract

**Objective:**

The objective of the present paper was to explore the clinical effect of one approach anterior decompression and fixation with posterior unilateral pedicle screw fixation for thoracolumbar osteoporosis vertebral compression fractures (OVCF).

**Methods:**

This is a single‐center retrospective analysis. A total of six thoracolumbar OVCF patients (four women and two men) with an average age of 65.2 years (58–72 years) who were treated between June 2016 and May 2018 were enrolled in the present study. The lesion segments included: 1 case at T11, 1 case at T12, 3 cases at L1, and 1 case at L2. The six thoracolumbar OVCF patients were treated with one approach anterior decompression and fixation with posterior unilateral pedicle screw fixation. After general anesthesia, patients were placed in the right lateral decubitus position, an approximately 10–15‐cm oblique incision was made along corresponding ribs, and the conventional left retroperitoneal and/or the extrapleural approach was performed for anterior lateral exposure. First, anterior decompression and fixation were performed, and then through the unilateral paraspinal muscle approach, posterior pedicle screw fixation was performed under the same incision. The back pain visual analogue scale (VAS), the Oswestry disability index (ODI), and the MacNab criteria were used to evaluate the clinical outcome. The radiographic analysis included the regional kyphosis angle and the fusion rate. Neurological status, operation time, intraoperative bleeding, the time of ambulation, hospital stay, and surgical complications were also assessed.

**Results:**

Surgery was successful in all six patients, who were followed up for 31.6 months (range, 23–46 months). The operation time was 125–163 min, with a median of 135 min. The preoperative blood loss was 580–1230 mL, with a median of 760 mL. The time of ambulation was 3–5 days, with a median of 4.2 days. The hospital stay was 8–15 days, with the median of 10.5 days. According to the Frankel classification of neurological deficits, of two patients with grade C preoperatively, one had improved to grade D and one had improved to grade E at final follow up; among four patients with grade D preoperatively, at the final follow up one remained the same and three had improved to grade E. The postoperative back pain VAS score decreased significantly, from 6.17 ± 0.75 preoperatively to 0.83 ± 0.41 postoperatively (*P* < 0.05). The mean ODI score was 73.7 ± 5.86 preoperatively and reduced to 21.85 ± 3.27 postoperatively (*P* < 0.05). According to the MacNab criteria, at the final follow up, two patients rated their satisfaction as excellent, three patients as good, and one patient as fair. The mean regional kyphosis angle was 22.17° ± 6.01°before surgery, which improved to 9.33° ± 3.88° at the final follow up (*P* < 0.05). At the final follow up, there were two patients who had achieved a grade 2 bony fusion (33.3%), three patients grade 3 (50.0%), and one patient grade 4 (16.7%). No incision infections, internal fixation failures or other complications were found during the perioperative and the follow‐up period.

**Conclusion:**

One approach anterior decompression and fixation with posterior unilateral pedicle screw fixation provides a novel method for thoracolumbar OVCF disease, with a satisfactory clinical outcome.

## Graphical abstract

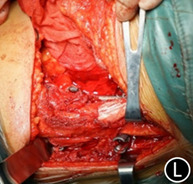

To treat thoracolumbar vertebral osteoporosis compression fractures, we applied one approach anterior decompression and fixation with posterior unilateral pedicle screw fixation. For this procedure, we placed the patient in the right lateral position, and we did not need to change the patient's position during the operation. Anterior pressure was thoroughly relieved, and fixation was performed without damaging the paravertebral muscles. Pedicle screws were placed through the paramedian muscle‐splitting approach, rebuilding the three‐column structure of the spine.

## Introduction

Almost 54 million Americans older than 50 years of age have osteoporosis^1^, with an estimated frequency of up to 2 million fractures annually^2^, and the rate of osteoporotic vertebral compression fractures (OVCF) is likely to increase due to the aging population. OVCF can be asymptomatic and is reported as an incidental finding in 15% of patients with radiologically documented fractures. Most symptomatic patients with OVCF pain improve within 3 months. Pain in many patients with OVCF often resolves spontaneously^3^. However, an estimated 33%^3^ of these patients have continued back pain, which can become debilitating and can be complicated with serious neurological deficit or paraplegia. These patients may be refractory to conservative care and may experience a substantial deterioration in their quality of life and a cascade of psychosocial disorders^4^. There are reported cases with lumbar compression fractures requiring bed rest for 26 days and thoracic compression fractures requiring bed rest for 13 days. The periods of limited activity were 159 days and 74 days, respectively^5^. In addition, OVCF has been shown to be related to a higher mortality rate at 10‐year follow up^6^. The thoracolumbar junction is the junction between the thoracic and the lumbar spine, composed of T11–L2. Spinal curvature changes from kyphosis to lordosis at the thoracolumbar junction, and the change from coronal to sagittal also occurs at this junction. Because of its specific position and composition, the thoracolumbar junction is usually affected in spinal fractures^7^.

Besides treatment of the underlying cause of the fracture, simple compression fractures (A1 or A3 according to the AO classification^8^) without neurological symptoms can be managed conservatively, such as with bed rest, taking medication or using a spinal brace, or surgically, through minimally invasive vertebroplasty or kyphoplasty^9^, ^10^, ^11^. However, failure during conservative or minimally invasive treatment of osteoporotic compression fractures may result in a severe deterioration of activity of daily living (ADL), with neurological deficits or progression of kyphosis, requiring surgical intervention. Since the first case of paraplegia due to OVCF treated with surgery was reported by Kempinsky *et al*.^12^, many studies have reported on various surgical techniques and outcomes for this condition, such as anterior decompression and fusion, posterior spinal shortening, posterior fixation with vertebroplasty, and combined anterior and posterior fixation^13^, ^14^, ^15^, ^16^. However, the ideal surgical procedure remains controversial. At present, posterior approach surgery is most commonly used due to its advantages in relation to fracture reduction, improvement in spinal stability, fewer complications, and surgeons being familiar with the posterior approach. Some authors have demonstrated that direct decompression of the middle column can be done through the posterior approach^17^. Nevertheless, with certain injuries, posterior approaches are limited in their ability to decompress the spinal canal, such as when severe vertebral body comminution or collapse occurs in association with large fracture fragments extending into the canal. Injuries above the L1 level represent another example because of the danger that exists due to the proximity to the spinal cord. Another limitation of the posterior approach is that it provides insufficient anterior support following a posterior distractive reduction for severe collapsed vertebral body, known as an “egg shell” deformity, which may result in loss of correction or implant failure^18^. Use of vertebroplasty or kyphoplasty procedures in combination with posterior short‐segment pedicle screw fixation in the treatment of acute thoracolumbar fractures has been reported to provide successful outcomes^19^, ^20^. However, in cases with osteoporosis, initial rigid stability has not been able to be achieved and postoperative kyphosis has subsequently progressed^15^, ^21^, ^22^. Furthermore, inadequate decompression was possible in patients with neurological deficit, and there was potential for neurological complications due to graft misplacement^23^. Thus, the validity of this approach as a surgical treatment option for osteoporotic vertebral fracture has not been established^24^.

In most OVCF patients, neurological deficit was caused by impact and compression to the ventral surface of the spinal cord. An anterior approach is advantageous for direct decompression as less manipulation of the injured cord and/or nerve roots is required. In addition, disc fragments can be completely excised and, from a biomechanical standpoint, strut grafting of the anterior and middle columns is favorable for obtaining a stable reconstruction and successful fusion. However, in patients with osteoporosis, an anterior vertebral body screw would be insufficient for initial stability because of the fragility of the vertebra^25^.

Therefore, combined with anterior spinal surgery^24^. Traditional combined anterior and posterior surgery requires changing the body position during the operation. There is a risk of spinal cord or nerve damage when changing position, and the duration of the operation is prolonged. Xia *et al*. (2009) reported on simultaneously combined anterior and posterior surgery for thoracolumbar fractures^18^. Posterior and anterior surgery were carried out with the patients remaining in the same position; it was found to be a reliable method that can achieve sufficient decompression, reduction, and reconstruction. However, it also required two incisions during the operation and the surgical trauma was considerable. OVCF patients were treated with one approach anterior decompression and fixation with posterior unilateral pedicle screw fixation. For this procedure, we place the patient in the right lateral position, and we do not need to change the patient's position during the operation. Anterior pressure was thoroughly relieved, and fixation was performed without damaging the paravertebral muscles. Pedicle screws were placed through the paramedian muscle‐splitting approach, rebuilding the three‐column structure of the spine. The purpose of this study was to: (i) explore the feasibility and advantages of one approach anterior decompression and fixation with posterior unilateral pedicle screw fixation for thoracolumbar osteoporotic vertebral compression fractures; (ii) analyze the clinical and radiological outcomes, and (iii) to summarize the main points and matters needing attention during the operation.

## Materials and Methods

### Inclusion Criteria and Exclusion Criteria

The inclusion criteria were: (i) patients with single segment thoracolumbar (T11–L2) osteoporotic vertebral compression fractures, with bone mineral density (BMD) measured by dual‐energy X‐ray absorptiometry at the lumbar vertebrae or femoral Ward area, and with T scores ≤−2.5 SD; (ii) patients with persistent lower back pain and neurological deficits in whom the symptoms were not significantly relieved after conservative treatment for 2 weeks, and who were treated with one approach anterior decompression and fixation with posterior unilateral pedicle screw fixation; (iii) main observation indicators included the low back pain visual analogue scale (VAS), the Oswestry disability index (ODI), neurological function, the regional kyphosis angle, and the fusion rate; and (iv) this was a single‐center retrospective analysis with follow‐up time longer than 1 year.

The exclusion criteria included: (i) patients who had sustained a violent fracture; (ii) patients with previous surgical history of the lesion segment or the adjacent segment; (iii) patients with spinal infection or tumor disease; and (iv) patients with severe underlying diseases who could not tolerate the operation.

### General Data

A total of four female and two male patients with an average age of 65.2 years (range, 58–72 years old) were enrolled in the study. The lesion segments were 1 case at T11, 1 case at T12, 3 cases at L1, and 1 case at L2. The reasons for fractures were: tumbles (3 cases), lifting (2 cases), and prolonged sitting (1 case); all of them experienced back pain, limited mobility, and nervous lesions; the mean preoperative back pain VAS score was 6.17. Neurologic status was graded using the Frankel classification system: two patients were classified as grade C and four patients as grade D.

The preoperative workup included posteroanterior and lateral X‐ray films, two‐dimensional CT, and MRI of the spine to assess the degree of neurological compression and kyphotic deformity. According to X‐ray findings, there were 2 cases with 25%–40% reduction of vertebral height (moderate deformity) and 4 cases with >40% reduction of vertebral height (severe deformity). The average regional kyphotic angle was 22.17° ± 6.01°. CT scans showed that the bone fragment entered the spinal canal, but the rate of bone fragment encroachment did not exceed 1/2 of the spinal canal, and there was no fracture in the spinous process, the lamina, or the adjacent segments. MRI scans found that the spinal cord or nerve were compressed by ventral bone fragments, but there was no signal change in the spinal cord. According to the AO classification system, all patients were considered A3 type.

### Surgical Procedure

#### Anesthesia and Position

All patients were treated with general anesthesia, placed in a standard right‐side position with a surgical bed waist bridge under the injury region; while their hip was kept from flexing, the lumbosacral and pubic symphysis were fixed with kickstands. Interoperative somatosensory evoked potentials (SEP) and motor evoked potentials (MEP) monitoring were used in all patients.

#### Approach and Exposure

An approximately 10–15‐cm oblique incision was made along corresponding ribs, and the conventional left retroperitoneal and/or the extrapleural approach was used for anterior lateral exposure. In general, T11 or T12 compression fractures were approached *via* the retroperitoneal and extrapleural routes and fractures at L1 or L2 were approached through the retroperitoneal route. Mobile C‐arm fluoroscopy was used to confirm the fracture level. To expose the L1 or L2 vertebral body, part of the psoas muscles were separated to expose the injured spine. A special retractor or two Kirschner wires inserted into the vertebral bodies were used to protect soft tissue during this procedure, and the segmental vessels were subsequently ligated.

#### Anterior Decompression and Fixation

The upper and lower adjacent discs were excised, followed by subtotal corpectomy. The vertebral canal fragments of the fragmented vertebral body or the disrupted disc were also carefully removed, but the posterior longitudinal ligament was preserved. Great care was taken to preserve as much of the bony endplates as possible during their preparation. After anterior decompression, anterior interbody fusion was performed using an anterior metal cage filled with cancellous bone chips harvested from the resected vertebral body, resected rib or iliac crest, and the inferior and superior ends of the cage were trimmed to match the sagittal alignment of the vertebral endplates. The surgical bed waist bridge was raised to expand the space between vertebral bodies, before implanting the cage. The cage was surrounded by autologous bone chips laterally and anteriorly. A rib graft was placed along the cage between the vertebrae when an extrapleural approach was used. If necessary, an iliac bone graft was used to fill the cage. The surgical bed waist bridge was restored, additional fixation was performed using two pedicle screws that were inserted into the lateral side of the proximal and distal vertebra of the fractured body. One rod was connected to the pedicle screws, and a careful reduction was attempted.

#### Posterior Fixation

Using the same incision, the posterior pedicle screws were inserted into the vertebral pedicles adjacent to the fractured vertebra through the unilateral paraspinal muscle approach. Then a rod was connected to the pedicle screws, and a careful reduction was attempted.

#### Drain and Postoperative Management

A drain was placed at anterior incision to prevent epidural hematoma after surgery and then the incision was sutured. Postoperatively, the loss through the drains was measured and recorded every day; the drain was removed when the blood loss was less than 50 mL per 24 h. Conventional dehydrate drugs and trophic nerve medicine were used to reduce the symptoms. All patients were braced postoperatively using a thoracolumbar sacral orthosis for 3 months and early ambulation was encouraged.

### Evaluation Methods and Indicators

#### Outcome Measurement

Neurological status was assessed using the Frankel score^26^, before surgery and at final follow up. The visual analogue scale (VAS score 0–10; 0, no pain; 10, the worst imagined) system was used to evaluate back pain control. The impact on the patient's daily life was assessed using the ODI, as described by Fairbank^27^. The VAS and ODI were evaluated preoperatively, at 3 months postoperatively, and at 12 months postoperatively. The MacNab criteria^28^ were used to evaluate clinical satisfaction at the final follow up. Operation time, intraoperative bleeding, time of ambulation, hospital stay, and surgical complications were also assessed.

(i) Frankel Functional Grade

The Frankel grade classification provides an assessment of spinal cord function and is used as a tool in spinal cord injury. The grades are as follows:

Grade A. Complete neurological injury: No motor or sensory function detected below level of lesion.

Grade B. Preserved sensation only: No motor function detected below level of lesion; some sensory function below level of lesion preserved.

Grade C. Preserved motor, nonfunctional: Some voluntary motor function preserved below level of lesion but too weak to serve any useful purpose; sensation may or may not be preserved.

Grade D. Preserved motor, functional: Functionally useful voluntary motor function below level of injury is preserved.

Grade E. Normal motor function: Normal motor and sensory function below level of lesion; abnormal reflexes may persist.

(ii) Visual Analogue Scale

The VAS was used to evaluate low back pain. Using a VAS ruler, the score was determined by measuring the distance (cm) on the 10‐cm line between the “no pain” anchor and the patient's mark, providing a range of scores from 0 to 10. A higher score indicated greater pain intensity. Patients described their low back pain intensity as between 0 (no pain) and 10 (worst pain ever).

(iii) Oswestry Disability Index

The ODI is a principal condition‐specific outcome measure used in the management of spinal disorders and to assess patient progress in routine clinical practice. The ODI score system includes 10 sections: pain intensity, personal care, lifting, walking, sitting, standing, sleeping, sex life, social life, and traveling. For each section of six statements, the total score is 5. Intervening statements are scored according to rank. If more than one box is marked in each section, the highest score is taken. If all 10 sections are completed, the score is calculated as follows: total scored out of total possible score × 100. If 1 section is missed (or not applicable), the score is calculated as: (total score/(5 × number of questions answered)) × 100%; 0%–20% is considered mild dysfunction, 21%–40% is moderate dysfunction, 41%–60% is severe dysfunction, and 61%–80% is considered as disability. For cases with a score of 81%–100%, they are either long‐term bedridden or exaggerating the impact of pain on their life.

(iv) MacNab Criteria

The patient satisfaction index (PSI), based on a modification of the MacNab criteria, was used. At each follow‐up visit and final follow up, patients were asked to select one of the following four possible choices: (i) “The surgery met my expectations, I have little pain, and I can perform desired activities with few limitations,” excellent; (ii) “The surgery met my expectations, I have occasional pain or sensory problems, but I can perform daily activities with minor limitations and do not take pain medication,” good; (iii) “The surgery met my expectations, my pain is somewhat improved, but I continue to need pain medication,” fair; and (iv) “My expectations were not met by the surgery; I am worse off or needed additional surgery,” poor. The PSI was dichotomized considering responses 1 through 3 as improved and response 4 as failed.

#### Radiological Data

The regional kyphosis angle was measured by lateral X‐ray preoperatively, at 3 days postoperatively, and at the final follow up. The fusion rate was measured by two‐dimensional CT at the final follow up.

(i) Regional Kyphotic Angle (°)

**Fig. 1:**
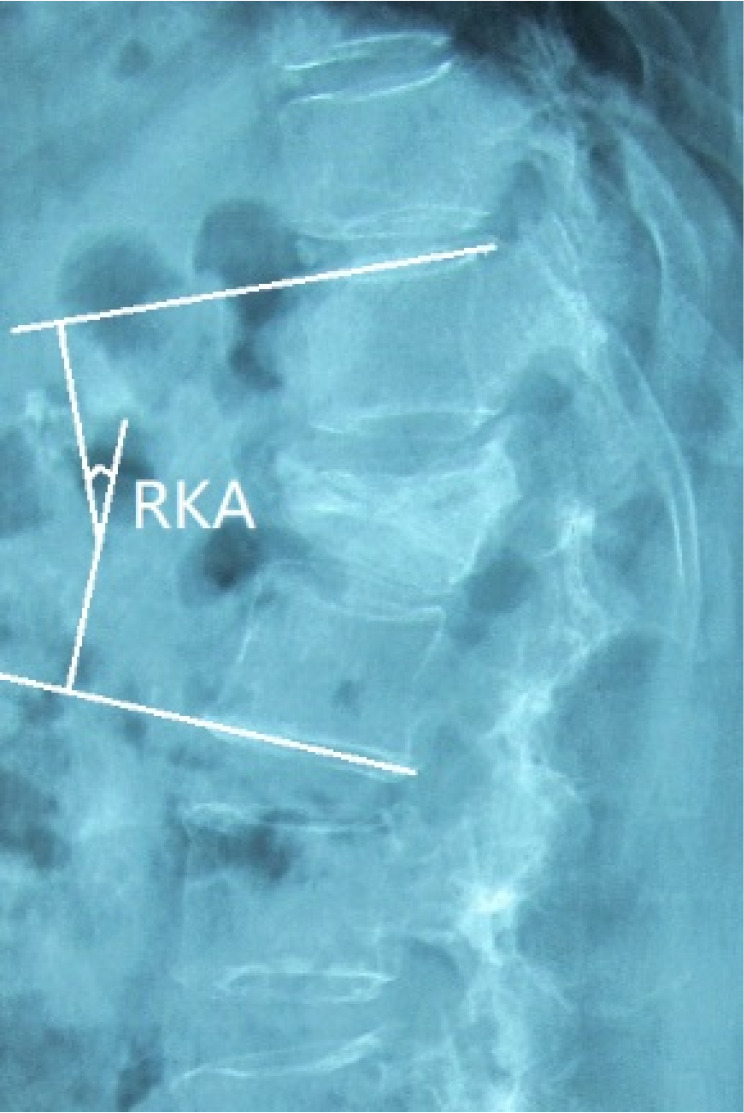
Regional kyphotic angle (RKA) was quantified using Cobb's angle, which was formed by lines drawn at the superior endplate of the vertebra above the fracture and inferior endplate of the vertebra below the fracture lateral radiograph.

The Cobb angle was measured on X‐ray films as the angle between the superior endplate of the vertebra above the fracture and the inferior endplate of the vertebra below the fracture (Fig. 1).

(ii) Fusion Rate

The fusion rate was divided into five grades: grade 0, no healing; grade 1, minimal consolidation of bone graft; grade 2, bone graft consolidation; grade 3, bridging callus; and grade 4, bridging callus with trabeculations^29^ (Table 1).

**TABLE 1:** Presence of fusion and extent of fusion based on grading scales used to evaluate CT images

Grade	Presence of fusion	Extent of fusion (%)
0	No healing	0 (not healed)
1	Minimal consolidation of bone graft	1–25 healed
2	Consolidation of bone graft	26–50 healed
3	Bridging callus	51–75 healed
4	Bridging callus with trabeculation	76–100 healed

### Statistical Analysis

**Fig. 2:**
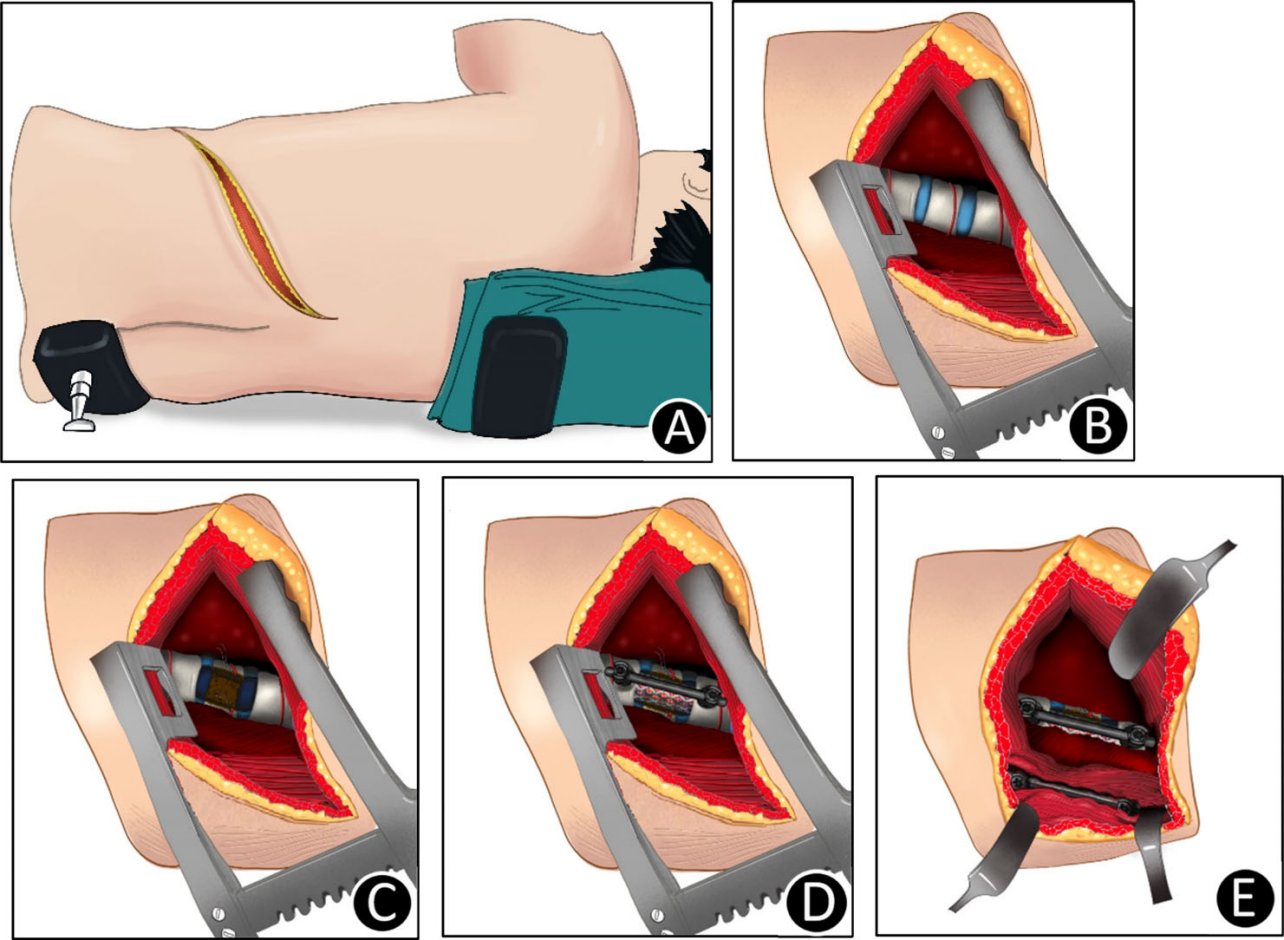
Schematic diagram of procedure. (A) The patient remains in the lateral decubitus position during the operation. An approximately 10–15‐cm oblique incision was made along corresponding ribs. (B) A conventional left retroperitoneal and/or extrapleural approach was performed for anterior lateral exposure. (C) Anterior decompression: The segmental vessels were ligated, and the upper and lower adjacent discs were excised, followed by subtotal corpectomy. (D) Anterior fixation: An anterior metal cage filled with cancellous bone chips were placed between the vertebral bodies, and pedicle screws were inserted into the vertebral bodies adjacent to the fracture vertebra, then a rod was connected to the pedicle screws. (E) Under the same incision, the posterior pedicle screws were inserted into the vertebral pedicles adjacent to the fracture vertebra through the unilateral paraspinal muscle approach, then a rod was connected to the pedicle screws.

**Fig. 3:**
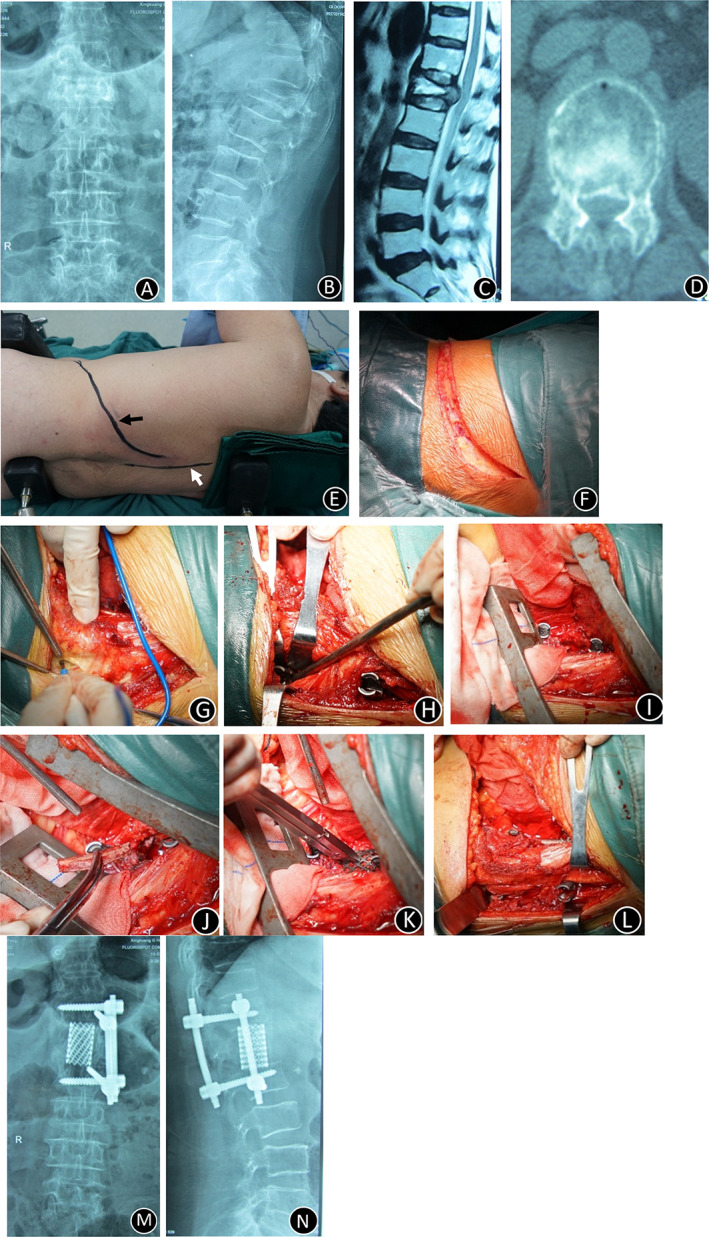
Clinical imaging from one representative patient (73‐year‐old woman) who complained of a lumbar 1 compression fracture. (A, B) X‐ray images of lumbar vertebrae anteroposterior and lateral position X‐ray films showed an L1 vertebral compression fracture. (C) T2‐weighted sagittal preoperative MRI of the lumbar spine showed that the spinal cord was compression by bone fragments. (D) The lumbar vertebrae CT indicates that the bone fragment had burst into the vertebral canal. (E) The patient remained in the lateral decubitus position during the operation; note the mark of the incision (black arrow) and the spinous process (white arrow). (F) An oblique incision was made along corresponding ribs. (G) After the anterior decompression was completed, the entry points of the posterior pedicle screws were exposed through the unilateral paraspinal muscle approach under the same incision. (H) The posterior pedicle screws were inserted into the vertebral pedicles adjacent to the fracture vertebra. (I) Two pedicle screws were inserted into the lateral side of the proximal and distal vertebras. (J) A rib graft was placed between the proximal and distal vertebras of the fractured vertebra. (K) An anterior metal cage filled with cancellous bone chips was implanted between the proximal and distal vertebras of the fractured vertebra. (L) Anterior fixation and posterior unilateral pedicle screw fixation is shown under one approach. (M,N) The postoperative X‐ray imaging indicated that the location of the internal fixator was excellent.

**Fig. 4:**
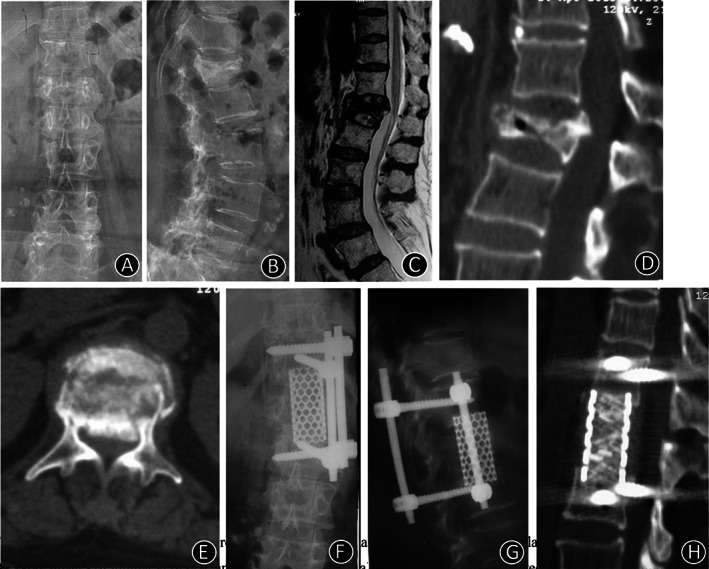
Clinical imaging from one representative patient (62‐year‐old woman) who complained of lumbar 1 compression fracture. (A, B) X‐ray images of lumbar vertebrae anteroposterior and lateral position X‐ray films showed L1 vertebral compression fracture. (C) T2‐weighted sagittal preoperative MRI of the lumbar spine showed that the spinal cord was compressed by a bone fragment. (D, E) The lumbar vertebrae CT indicates that the bone fragment had burst into the vertebral canal. (F, G) At 12 months postoperatively, the X‐ray imaging indicated that the location of the internal fixator was excellent. (H) At 12 months postoperatively, two‐dimensional CT indicated that the auto graft bone fusion was excellent.

**Fig. 5:**
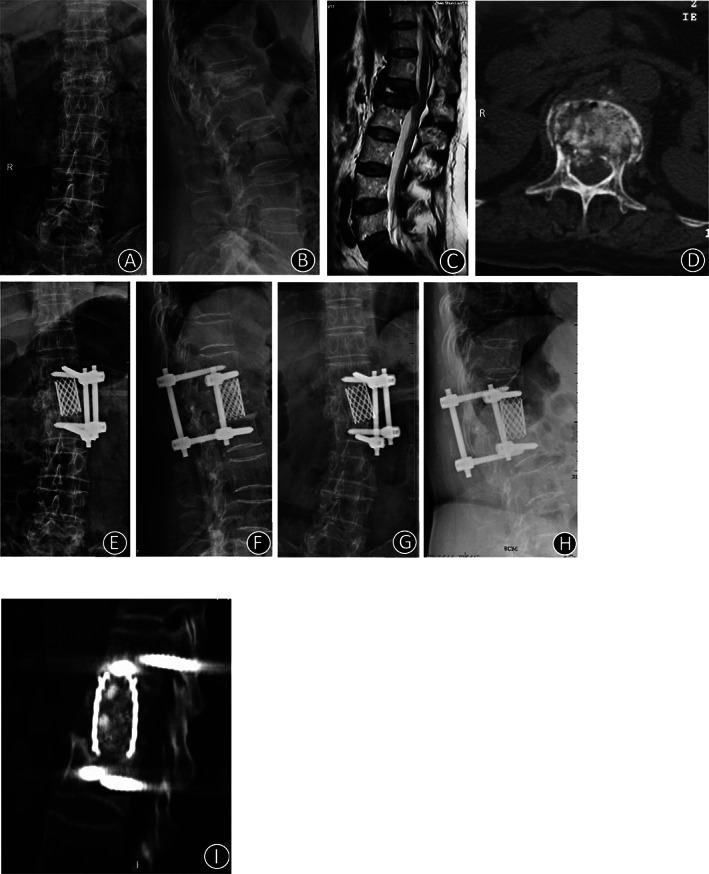
Clinical imaging from one representative patient (71‐year‐old woman) who complained of lumbar 1 compression fracture. (A, B) X‐ray images of lumbar vertebrae anteroposterior and lateral position X‐ray films showed L1 vertebral compression fracture. (C) T2‐weighted sagittal preoperative MRI of the lumbar spine showed that the spinal cord was compressed by the bone fragment. (D) The lumbar vertebrae CT indicates that the bone fragment had burst into vertebral canal. (E, F) At 3 days postoperatively, the X‐ray imaging indicated that the location of the internal fixator was excellent. (G,H) At 12 months postoperatively, the X‐ray imaging indicated that the location of the internal fixator was excellent. (I) At 12 months postoperatively, two‐dimensional CT indicates that the auto graft bone fusion was excellent.

Statistical analysis on all parameters was performed using SPSS17.0. software (SPSS, Chicago, IL, USA). Data were expressed as mean and standard deviation (mean ± SD) and were compared using one‐way analysis of variance (one‐way ANOVA). All *P‐*values ≤0.05 were considered statistically significant. According to the degree of vertebral compression, we divided the included patients into two subgroups, moderate deformity and severe deformity, and performed subgroup analysis. We used one‐way ANOVA to perform between‐group and within‐group analysis for the VAS score, the ODI score, and the Cobb angle, respectively (Figs 2, 3, 4, 5).

## Result

### General Characteristics

Surgery was successful in all six patients, and they were followed up for 23–46 months, with a median of 31.6 months. Follow up was conducted by telephone survey and periodic outpatient visits.

The operation time was 125–163 min, with a mean of 135 min; the preoperative blood loss was 580 – 1230 mL, with a mean of 760 mL; the time of ambulation was 3–5 days, with a mean of 4.2 days; and the hospital stay was 8–15 days, with a mean of 10.5 days.

### Clinical Outcomes

#### Neurological Evaluation

Of the two patients classified preoperatively as grade C according to Frankel classification of neurological deficits, at the final follow up one had improved to grade D and the other to grade E. Among the four patients classified as grade D preoperatively, at the final follow up one remained the same and the other three had improved to grade E (Table 2).

**TABLE 2:** Demonstrating the neurological Frankel grades of patients before surgery and at final follow‐up

Preoperative	Postoperative				
	A	B	C	D	E
A					
B					
C				1	1
D				1	3
E					

#### Visual Analogue Scale

The postoperative back pain VAS score decreased significantly, from 6.17 ± 0.75 preoperatively to 1.33 ± 0.82 and 0.83 ± 0.41 postoperatively at 3 months and 12 months, respectively (*P* < 0.05). Compared with preoperative data, the back pain VAS score at 3 months postoperatively had decreased by 78.4% and at 12 months postoperatively had decreased by 86.5%. The VAS score at 12 months postoperatively was lower than that at 3 months postoperatively, but the difference was not statistically significant (*P* > 0.05) (Table 3). In the moderate deformity group, the back pain VAS score was 1.00 ± 0.00 at 3 months postoperatively and 0.50 ± 0.71 at 12 months postoperatively; these values were significantly lower than that recorded preoperatively (6.00 ± 1.41; *P* < 0.05). In the severe deformity group, the back pain VAS score was 1.50 ± 1.00 at 3 months postoperatively and 1.00 ± 0.00 12 months postoperatively; these values were significantly lower than that recorded preoperatively (6.25 ± 0.50; *P* < 0.05). However, the VAS score had no significant difference between the groups preoperatively and postoperatively (*P* > 0.05).

**TABLE 3:** Preoperative and postoperative VAS and ODI scores in patients who underwent the operation

	Preoperative	3 months	12 months
VAS	6.17 ± 0.75	1.33 ± 0.82	0.83 ± 0.41
*P*‐value		0.000* 0.224^†^	0.000*
ODI score	73.7 ± 5.86	25.93 ± 2.69	21.85 ± 3.27
*P*‐value		0.000* 0.112^†^	0.000*

ODI, Oswestry disability index; VAS, visual analogue scale.

* Compared with preoperative data,

^†^ Compared with 12 months postoperation,

#### Oswestry Disability Index

The ODI score was 73.7 ± 5.86 preoperatively and reduced significantly to 25.93 ± 2.69 and 21.85 ± 3.27 postoperatively at 3 months and 12 months, respectively (*P* < 0.05). Compared with preoperative data, the ODI score at 3 months postoperatively had decreased by 64.8% and at 12 months postoperatively had decreased by 70.4%. The ODI score at 12 months postoperatively was lower than that at 3 months postoperatively, but the difference was not statistically significant (*P* > 0.05) (Table 3).

In the moderate deformity group, the ODI score was 27.78 ± 1.57 at 3 months postoperatively and 22.22 ± 3.14 at 12 months postoperatively; these values were significantly lower than that recorded preoperatively (78.88 ± 4.70; *P* < 0.05). In the severe deformity group, the ODI score was 25.00 ± 2.80 at 3 months postoperatively and 21.67 ± 3.80 at 12 months postoperatively; these values were significantly lower than that recorded preoperatively (71.11 ± 4.80; *P* < 0.05). The ODI score was not significantly different between the groups preoperation and postoperation (*P* > 0.05).

#### MacNab Criteria

According to the MacNab criteria, at the final follow up two patients were assessed as excellent (33.3%), three patients as good (50%), and one patient as fair (16.7%). The overall success rate was 83.3%, and the symptomatic improvement was 100%.

### Radiological Data

#### Regional Kyphosis Cobb Angle

The kyphosis angle was 22.17° ± 6.01° before surgery and reduced significantly to 7.5 ± 3.27° and 9.33° ± 3.88° at 3 days after surgery and at the final follow up, respectively (*P* < 0.05). At final follow up, there was 12.8°4 correction in kyphosis compared to that before surgery and 1.83° loss of correction compared to that at the initial postoperative radiograph (Table 4).

**TABLE 4:** Preoperation and postoperation regional kyphosis Cobb angle

	Preoperative	3 days	Final follow‐up
Cobb angle	22.17° ± 6.01°	7.5° ± 3.27°	9.33° ± 3.88°
*P*‐value		0.000* 0.495^†^	0.000*

* Compared with preoperation.

^†^ Compared with final follow up.

In the moderate deformity group, the kyphosis angle was 23.00° ± 5.66° before surgery, 8.50° ± 2.12° 3 days after surgery, and 9.50° ± 3.54° at the final follow up. The postoperative kyphosis angle was significantly lower than that before surgery (*P* < 0.05). In the severe deformity group, the kyphosis angle was 21.75° ± 6.99° before surgery, 7.00° ± 3.92° 3 days after surgery, and 9.25° ± 4.57° at the final follow up. The postoperative kyphosis angle was significantly lower than that before surgery (*P* < 0.05). The kyphosis angle had no significant difference between the groups before and after surgery (*P* > 0.05).

#### Fusion Rate

At the final follow up, two patients had achieved grade 2 bony fusion (33.3%), three patients grade 3 (50.0%), and one patient grade 4 (16.7%). No grade 0 healing was observed.

In the moderate deformity group, one patient achieved grade 2 bony fusion, and one patient reached grade 3. In the severe deformity group, one patient achieved grade 2 bony fusion, two patients grade 3, and one patient grade 4.

## Discussion

Because of bone loss and trabecula decrease leading to bone fragility and variation in bone tissue, thoracolumbar vertebral compression can easily occur in patients with osteoporosis. Thoracolumbar vertebral compression patients without spinal stenosis can typically be treated with conservative treatment or minimally invasive surgery (percutaneous vertebroplasty or and percutaneous kyphoplasty); in contrast, patients with severe vertebral compression fractures and neurological symptoms always need to be treated with surgery^30^.

Posterior surgery has the advantage of facilitating realignment of the spinal column. Some authors have confirmed that direct decompression of the middle column can be performed through the posterior approach^17^. However, adequate direct neural decompression cannot be achieved, and unacceptably high failure rates have been reported when traditional short‐segment pedicle screw fixation is performed alone^31^. In most patients with thoracolumbar fractures, neurological deficit was caused by impact and compression to the ventral surface of the spinal cord. Anterior decompression surgery has the advantage of directly decompressing neural elements but may result in further collapse of the vertebral column due to difficulty in obtaining rigid fixation^25^, ^32^. In addition, in elderly patients, because of the decreased strength of the posterior ligament complex and minimal stability, anterior grafts alone are not adequate. Therefore, patients with osteoporosis and thoracolumbar vertebral compression fractures always need posterior combined with anterior spinal surgery^24^.

Numerous previous biomechanical studies have attempted to comparatively evaluate the unilateral and bilateral pedicle screw fixation approaches. Chen *et al*. demonstrated that unilateral pedicle screw (UPS) fixation was adequate to maintain the stability of the spine in a biomechanics study^33^. Similar studies have confirmed that the UPS system is effective in reducing stress shielding of the vertebra and diminishing peak stress arising in the adjacent levels above and below the fusion. In addition, UPS fixation resulted in a lower incidence of adjacent‐segment degeneration than bilateral pedicle screw fixation^34^. We also performed some biomechanical testing to determine whether the two fixed methods could attain the same mechanical stability in treating lumbar degenerative disease^35^, ^36^, ^37^.

There has been a report of simultaneously combined anterior and posterior surgery for thoracolumbar fractures: Posterior and anterior surgery were carried out with the patients remaining in the same position^18^. We applied the unilateral fixation technique in the anterior and posterior combined surgery. The operation was performed under the same incision and *via* the paravertebral muscle approach, which could reduce the damage to the posterior soft tissue. It is not necessary to change the patient's position during the operation. Not having to do so can shorten the operation time and prevent iatrogenic injury from changing position. In our study, the operation time was 125–163 min, with the median of 135 min. The preoperative blood loss was 580–1230 mL, with a median of 760 mL. The operative time and preoperative blood loss were lower than in previously reported anterior–anterior combined surgeries^18^, ^24^.

This procedure combined the advantages of anterior and posterior surgery. It achieved good reduction, complete decompression, and rigid fixation at the same time. In our study, the postoperative back pain VAS score decreased significantly, from 6.17 ± 0.75 preoperatively to 0.83 ± 0.41 at 12 months postoperatively. The mean ODI score was 73.7 ± 5.86 preoperatively and reduced significantly to 21.85 ± 3.27 at 12 months postoperatively. The neurological function of the patients was significantly improved. Moreover, there was no loose or displaced internal fixation was found at follow up, and the fusion of bone grafting was good, which was conducive to the reconstruction of the spinal stability, restoration of normal sequence. There was no significant difference in clinical efficacy between the moderate deformity group and the severe deformity group, indicating that this technique can achieve satisfactory clinical efficacy in both moderate and severe deformity thoracolumbar osteoporotic vertebral compression fractures. Therefore, this operation has the advantages of satisfactory clinical efficacy, short operation time, less intraoperative blood loss, firm internal fixation, and fewer postoperative complications. This procedure is effectively for treating thoracolumbar osteoporotic vertebral compression fractures.

In the process of the removing vertebral bodies and decompression, bleeding increased, so after removing the injured vertebral segments through the retroperitoneal approach, hemostasis with performed with provisional gauze. We could place pedicle screws through the paraspinal muscles into the adjacent vertebral bodies for temporary fixation, which could prevent instability leading to spinal cord secondary damage. We placed pedicle screws into adjacent vertebral bodies before the spinal cord decompression to reduce the decompression time as much as possible. The forepart of the vertebral body was removed by osteotome. When close to the vertebral posterior wall, we could scrape the vertebral wall carefully with a multi‐angle curet, which could reduce the risk of spinal cord injury. Most surgeons view intraoperative electromyography (EMG) as an important adjunct to improve patient safety during spinal surgery, and EMG could be used to reduce the incidence of nerve injury^38^, ^39^. In our operation, we applied interoperative SEP and MEP monitoring during the operation, and no intraoperative nerve injuries occurred. The segmental vessel of the injured vertebra was ligated during the operation, and the segmental vessels of the adjacent vertebral body do not usually need special treatment. When treating the segment vessel of the injured vertebra, first we exposed and dissociated the vessel clearly, and then we clamped the vessel close to the front and back of the vertebral body with two right‐angle clamps. Finally, the blood vessel was cut off with an electric knife and double stitched. To avoid loss of vertebral body height and kyphosis appearing after the operation, the length of the titanium mesh should be appropriately. The waist bridge was shaken before implanting the titanium mesh, and the waist bridge was restored after implanting the titanium mesh. Pressure was exerted with the posterior pedicle screw rod system and the rib was placed in front of or to the left of the titanium mesh to increase the fusion rate.

### Conclusion

The posterior and anterior operation could be completed with the patient in the same position. There is no need to change the position of the body during the operation, hence reducing the operation time. The pedicle screws were placed through the spatium intermusculare, which could reduce injury to the posterior ligament complex and maintain the stability of the spine. Combining posterior and anterior operations to reconstruct the three‐column structure is more consistent with the biomechanics of the spine and is conducive to early rehabilitation and functional exercise. One approach anterior decompression and fixation with posterior unilateral pedicle screw fixation is an effective method for treating thoracolumbar osteoporosis vertebral compression fractures, with a short operation time, little trauma, a satisfactory curative effect, and quick recovery.

Compared with simple anterior or posterior operations, this technique is more invasive and requires strict control of the indication. It is suitable for patients with spinal fractures whose fracture blocks enter the spinal canal and cause nerve damage. Performing anterior decompression is necessary, but due to osteoporosis, simple anterior fixation is not enough, and auxiliary posterior fixation is needed. Our study is limited by the small sample size. Future studies with larger sample sizes are needed to further confirm our conclusion.
